# Supplementary material for: Live-cell single-molecule dynamics of PcG proteins imposed by the DIPG H3.3K27M mutation
Source: Nat Commun. 2018 May 25;9:2080. doi: 10.1038/s41467-018-04455-7 (PMC5970213; doi:10.1038/s41467-018-04455-7)
Supplement: Supplementary file 1 — Supplementary Information [file 41467_2018_4455_MOESM1_ESM.pdf]

SUPPLEMENTARY INFORMATION

Live-cell Single-Molecule Dynamics of PcG Proteins Imposed by the DIPG H3.3K27M Mutation

'Tatavosian et al.'

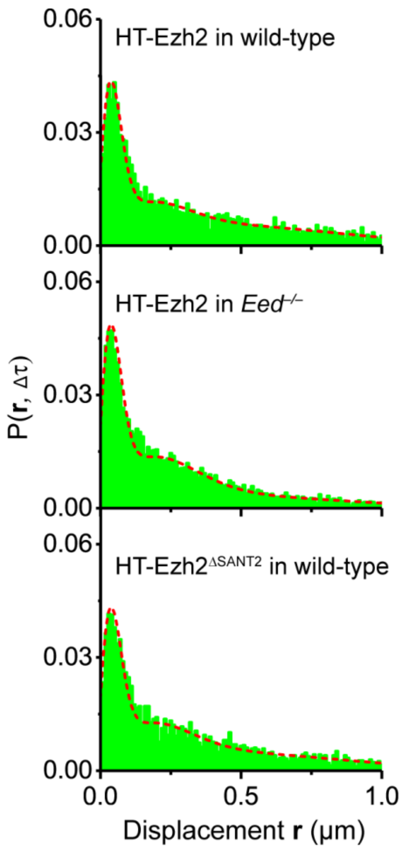

Supplementary Figure 1 (relative to Figure 3). Single-molecule displacement histograms for dissecting the effects of the core components Eed and Suz12 of PRC2 on the binding and search mechanism of Ezh2.

Single-molecule displacement histograms for HaloTag-Ezh2 in wild-type mES cells replicated from Figure 1c, for HaloTag-Ezh2 in *Eed*$^{-/-}$ mES cells, and for HaloTag-Ezh2$^{\Delta SANT2}$ in wild-type mES cells.

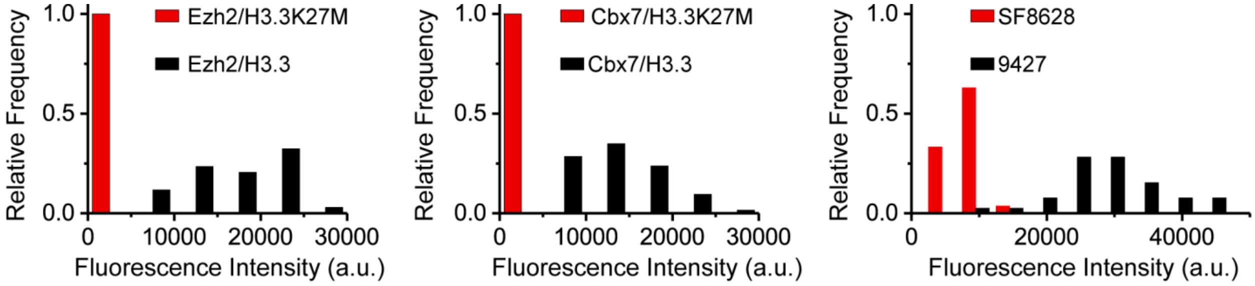

Supplementary Figure 2 (relative to Figure 4). **Effects of the DIPG H3.3K27M mutation on the H3K27me3 level.**

We quantified the H3K27me3 level in cells carrying *H3.3* or *H3.3K27M* by immunofluorescence. The fluorescence intensity of individual cells was quantified by ImageJ. The distribution of fluorescence intensities is shown. a.u., arbitrary unit.

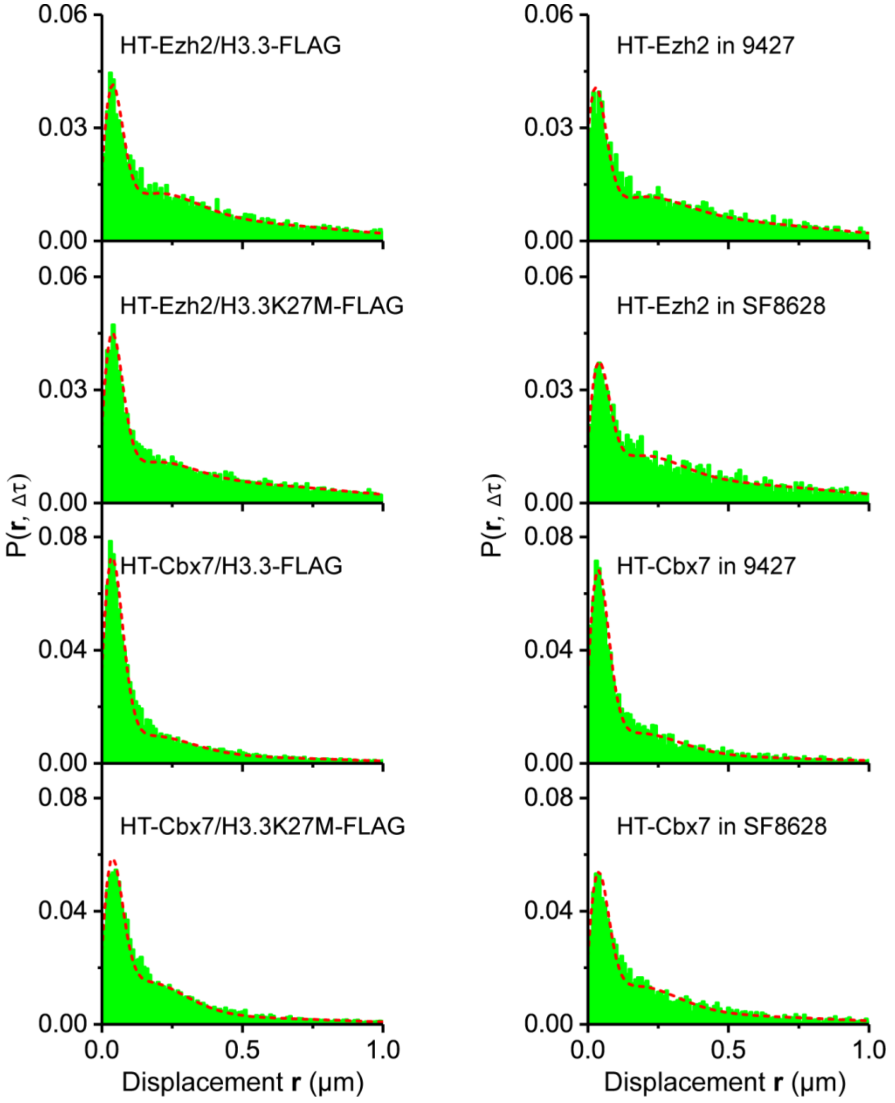

Supplementary Figure 3 (relative to Figure 4). **Single-molecule displacement histograms for studying the effects of the DIPG H3.3K27M mutation on the kinetic fraction and diffusion constant of Ezh2 and Cbx7.**

Single-molecule displacement histograms for HaloTag-Ezh2 in HEK293T cells expressing *H3.3-FLAG* or *H3.3K27M-FLAG*, and for HaloTag-Cbx7 in HEK293T cells expressing *H3.3-FLAG* or *H3.3K27M-FLAG*, for HaloTag-Ezh2 in 9427 and SF8628 cells, and for HaloTag-Cbx7 in 9427 and SF8628 cells.

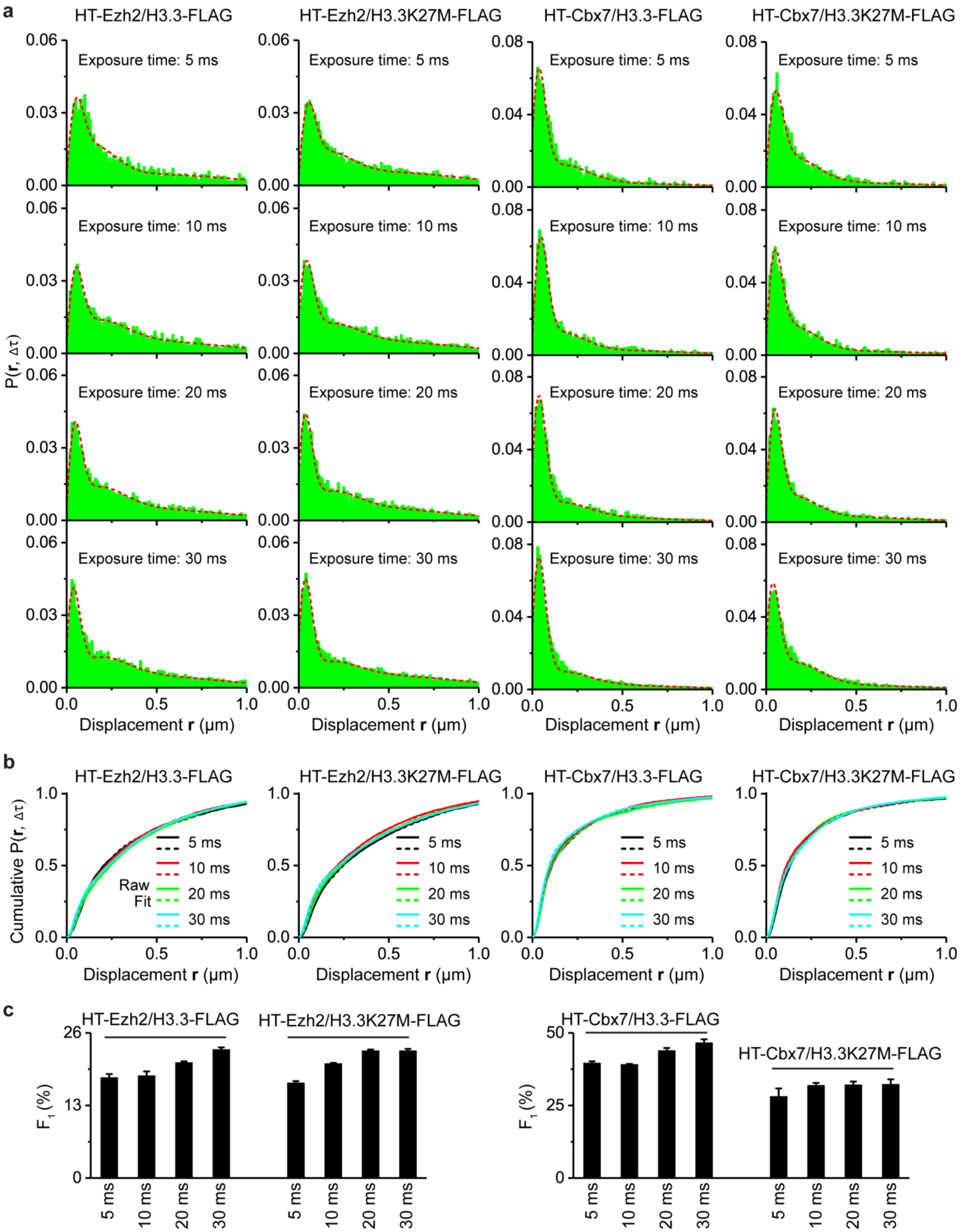
a
HT-Ezh2/H3.3-FLAG
HT-Ezh2/H3.3K27M-FLAG
HT-Cbx7/H3.3-FLAG
HT-Cbx7/H3.3K27M-FLAG
Exposure time: 5 ms
Exposure time: 10 ms
Exposure time: 20 ms
Exposure time: 30 ms
P(r, Δτ)
Displacement r (μm)
b
HT-Ezh2/H3.3-FLAG
HT-Ezh2/H3.3K27M-FLAG
HT-Cbx7/H3.3-FLAG
HT-Cbx7/H3.3K27M-FLAG
Cumulative P(r, Δτ)
Raw
Fit
5 ms
10 ms
20 ms
30 ms
Displacement r (μm)
c
HT-Ezh2/H3.3-FLAG
HT-Ezh2/H3.3K27M-FLAG
HT-Cbx7/H3.3-FLAG
HT-Cbx7/H3.3K27M-FLAG
F1 (%)
5 ms
10 ms
20 ms
30 ms

Supplementary Figure 4 (relative to Figure 4). **Effects of the exposure time on the kinetic fraction and diffusion constant of Ezh2 and Cbx7 in HEK293T cells expressing *H3.3* or *H3.3K27M*.**

(a) Single-molecule displacement histograms for HaloTag-Ezh2 in HEK293T cells expressing *H3.3-FLAG* (exposure time 5 ms: N = 49 cells, n = 4171 displacements; exposure time 10 ms: N = 76 cells, n = 6383 displacements; exposure time 20 ms: N = 40 cells, n = 6923 displacements; and exposure time 30 ms: N = 54 cells, n = 9034 displacements) or *H3.3K27M-FLAG* (exposure time 5 ms: N = 86 cells, n = 8239 displacements; exposure time 10 ms: N = 66 cells, n = 7790 displacements; exposure time 20 ms: N = 72 cells, n = 10276 displacements; and exposure time 30 ms: N = 127 cells, n = 22273 displacements), and for HaloTag-Cbx7 in HEK293T cells expressing *H3.3-FLAG* (exposure time 5 ms: N = 43 cells, n = 2600 displacements; exposure time 10 ms: N = 65 cells, n = 4181 displacements; exposure time 20 ms: N = 66 cells, n = 5977 displacements; and exposure time 30 ms: N = 78 cells, n = 14101 displacements) or *H3.3K27M-FLAG* (exposure time 5 ms: N = 42 cells, n = 2680 displacements; exposure time 10 ms: N = 54 cells, n = 5107 displacements; exposure time 20 ms: N = 50 cells, n = 7927 displacements; and exposure time 30 ms: N = 89 cells, n = 15755 displacements).

(**b**) Cumulative distribution of single-molecule displacements for HaloTag-Ezh2 or HaloTag-Cbx7 in HEK293T cells expressing *H3.3-FLAG* or *H3.3K27M-FLAG*. The distributions were decomposed into three populations. Fitted parameters are shown in Supplementary Table 5.

(**c**) Fraction of the chromatin-bound population (F1) for HaloTag-Ezh2 or HaloTag-Cbx7 in HEK293T cells expressing *H3.3-FLAG* or *H3.3 K27M-FLAG*. Results are means ± SD of three biological replicates.

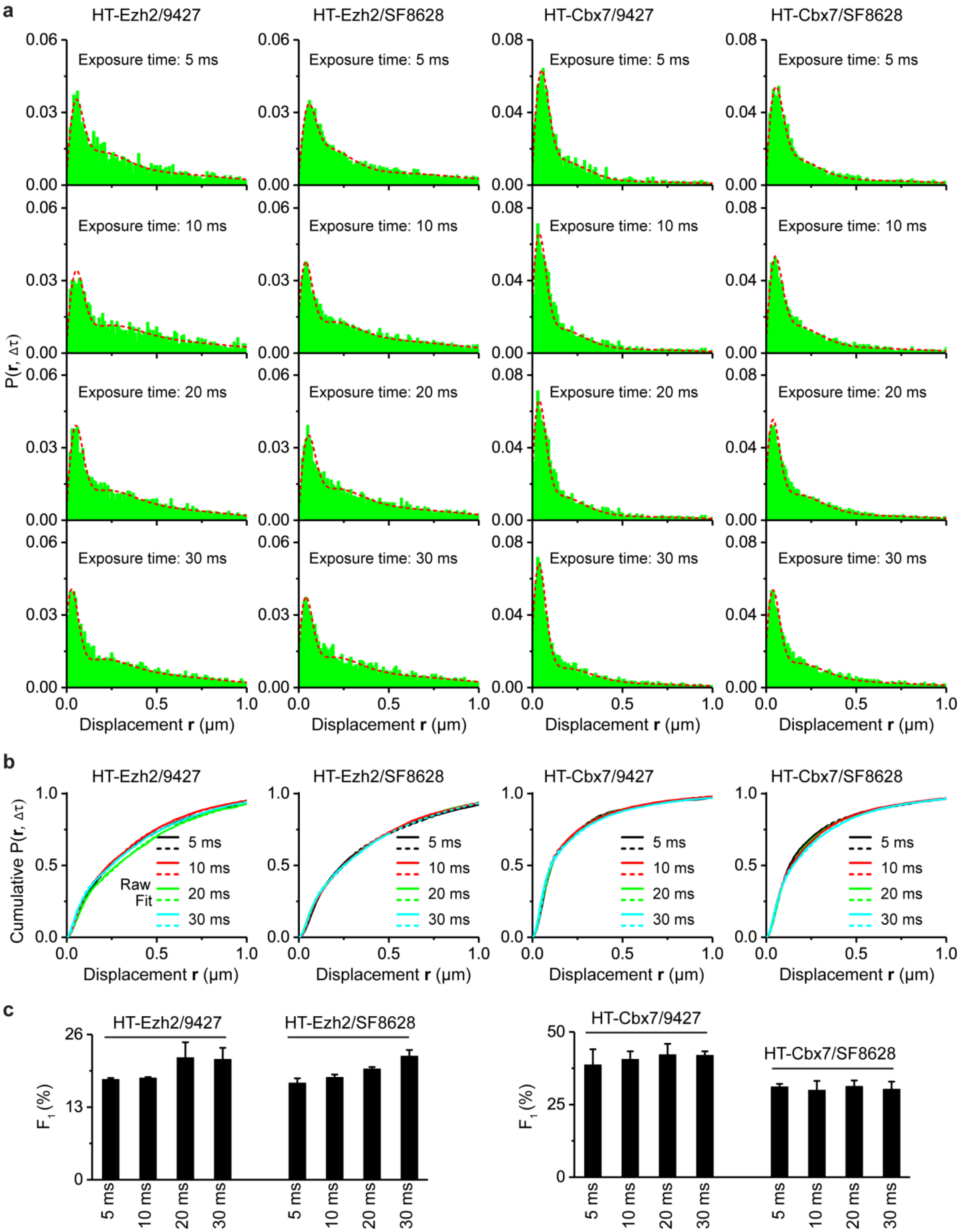

a
HT-Ezh2/9427
HT-Ezh2/SF8628
HT-Cbx7/9427
HT-Cbx7/SF8628
Exposure time: 5 ms
Exposure time: 10 ms
Exposure time: 20 ms
Exposure time: 30 ms
P(r, Δτ)
Displacement r (μm)
b
Cumulative P(r, Δτ)
5 ms
10 ms
20 ms
30 ms
Raw
Fit
c
F1 (%)

Supplementary Figure 5 (relative to Figure 4). **Effects of the exposure time on the kinetic fraction and diffusion constant of Ezh2 and Cbx7 in 9427 and SF8628 cells.**

(a) Single-molecule displacement histograms for HaloTag-Ezh2 in 9427 (exposure time 5 ms: N = 49 cells, n = 3343 displacements; exposure time 10 ms: N = 52 cells, n = 3751 displacements; exposure time 20 ms: N = 66 cells, n = 5731 displacements; and exposure time 30 ms: N = 73 cells, n = 6806 displacements) or SF8628 (exposure time 5 ms: N = 81 cells, n = 9580 displacements; exposure time 10 ms: N = 72 cells, n = 9285 displacements; exposure time 20 ms: N = 57 cells, n = 7032 displacements; and exposure time 30 ms: N = 43 cells, n = 6900 displacements) cells, and for HaloTag-Cbx7 in 9427 (exposure time 5 ms: N = 57 cells, n = 2901 displacements; exposure time 10 ms: N = 62 cells, n = 4184 displacements; exposure time 20 ms: N = 76 cells, n = 6281 displacements; and exposure time 30 ms: N = 37 cells, n = 5280 displacements) or SF8628 (exposure time 5 ms: N = 71 cells, n = 6336 displacements; exposure time 10 ms: N = 63 cells, n = 7011 displacements; exposure time 20 ms: N = 66 cells, n = 10249 displacements; and exposure time 30 ms: N = 32 cells, n = 8181 displacements) cells.

(**b**) Cumulative distribution of single-molecule displacements for HaloTag-Ezh2 or HaloTag-Cbx7 in 9427 or SF8628 cells. The distributions were decomposed into three populations. Fitted parameters are shown in Supplementary Table 5.

(**c**) Fraction of the chromatin-bound population (F1) for HaloTag-Ezh2 or HaloTag-Cbx7 in 9427 or SF8628 cells. Results are means ± SD of three biological replicates.

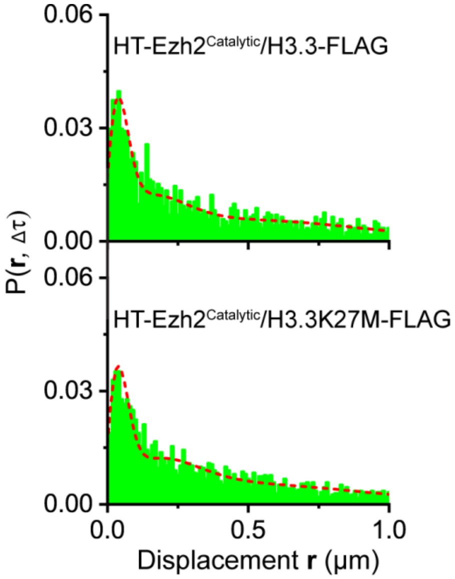

Supplementary Figure 6 (relative to Figure 6). **Single-molecule displacement histograms for studying the effects of H3.3K27M on the residence time of Ezh2^{Catalytic} on chromatin.**

Single-molecule displacement histograms for HaloTag-Ezh2^{Catalytic} in HEK293T cells expressing *H3.3-FLAG* or *H3.3K27M-FLAG*.

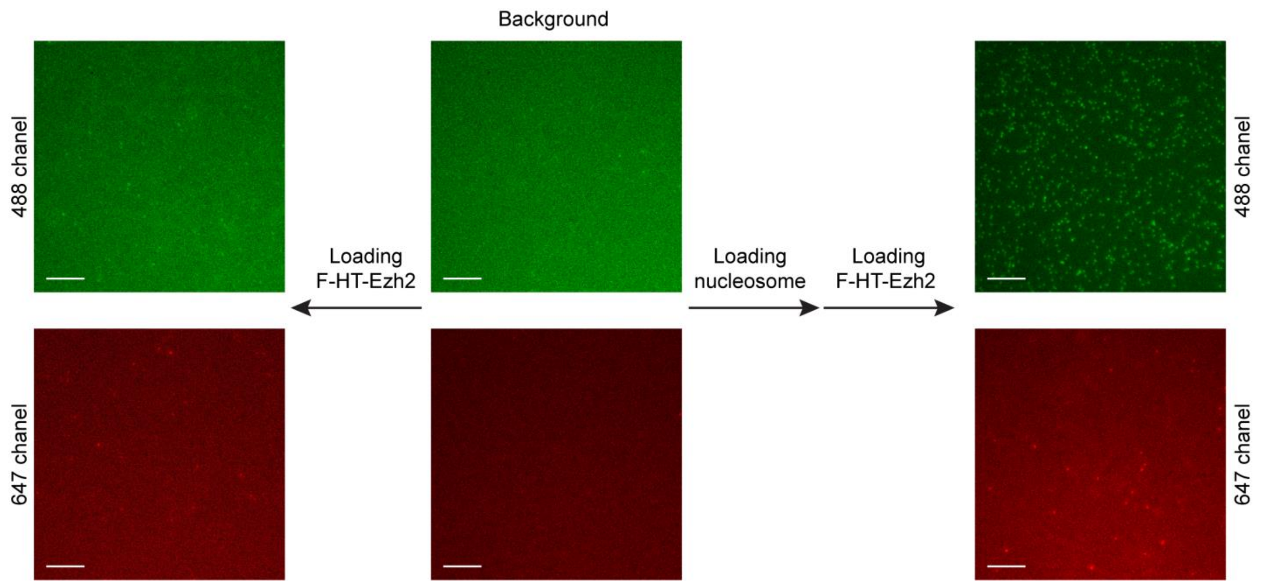

Supplementary Figure 7 (relative Figure 6). **Control experiments for *in vitro* single-molecule binding assay.**

The nucleosomal position was recorded by the 488 channel and FLAG-HaloTag-Ezh2 by the 647 channel. The middle panel shows the image of the quartz coverslip of the flow cell, which has been passivated and functionalized by NeutrAvidin. The left panel shows the image of the quartz coverslip loaded with FLAG-HaloTag-Ezh2 labelled with JF_{646}. The right panel shows the image of the quartz coverslip loaded first with nucleosome labelled with Biotin and Alexa Fluor® 488 and then with FLAG-HaloTag-Ezh2 labelled with JF_{646}.

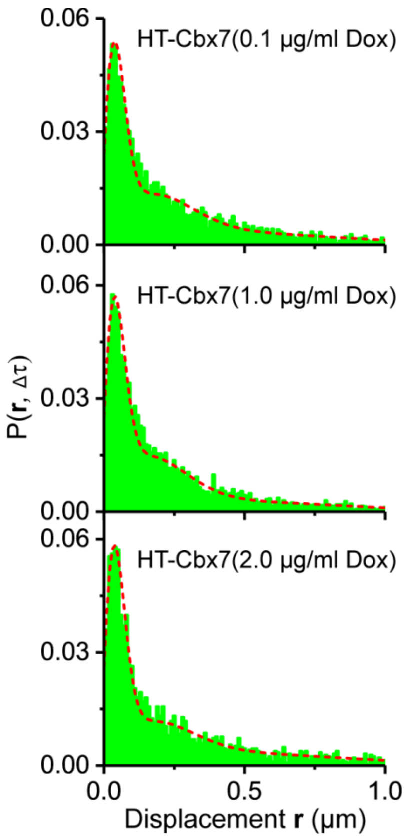

Supplementary Figure 8 (relative to Figure 8). **Single-molecule displacement histograms for analyzing the effects of increasing HaloTag-Cbx7 levels on the kinetic fraction and diffusion constant in SF8628 cells.**

Single-molecule displacement histograms for HaloTag-Cbx7 in SF8628 cells administrating 0.1 μg per ml, 1.0 μg per ml and 2.0 μg per ml of doxycycline (Dox).

Supplementary Table 1 (Relative to Figure 1). Kinetic fractions and diffusion constants.

		F_1	D_1 ($\mu m^2 s^{-1}$)	F_2	D_2 ($\mu m^2 s^{-1}$)	F_3	D_3 ($\mu m^2 s^{-1}$)
Control experiments	H2A-HT in wild-type	(82 ± 2)%	0.0001	NA	NA	(18 ± 2)%	5.0 ± 0.1
	HT-NLS in wild-type	(8 ± 1)%	0.0001	(20 ± 1)%	0.32 ± 0.02	(72 ± 2)%	5.0 ± 0.1
	HT-Cbx7 in wild-type	(40 ± 2)%	0.0001	(40 ± 1)%	0.60 ± 0.10	(20 ± 3)%	4.1 ± 0.1
HT-Ezh2	HT-Ezh2 in wild-type	(23 ± 1)%	0.0001	(23 ± 4)%	0.54 ± 0.09	(54 ± 5)%	4.2 ± 0.4
	HT-Ezh2 in *Ezh2*$^{-/-}$	(22 ± 1)%	0.0001	(23 ± 1)%	0.48 ± 0.09	(55 ± 8)%	4.2 ± 0.1
HT-Eed	HT-Eed in wild-type	(20 ± 1)%	0.0001	(29 ± 4)%	0.52 ± 0.07	(51 ± 3)%	4.4 ± 1.2
	HT-Eed in *Eed*$^{-/-}$	(22 ± 1)%	0.0001	(26 ± 2)%	0.54 ± 0.10	(52 ± 2)%	4.6 ± 0.2

Supplementary Table 2 (Relative to Figure 2). Search mechanism parameters.

		F_{1tb}	F_{1sb}	τ_{tb} (s)	τ_{sb} (s)	N_{trial}	τ_{3D} (s)	τ_{search} (s)
HT-Ezh2	HT-Ezh2 in wild-type	(18 ± 1)%	(4.6 ± 0.3)%	0.9 ± 0.1	10.0 ± 0.2	5.0 ± 0.4	41.6 ± 5.2	210 ± 19
	HT-Ezh2 in *Ezh2*$^{-/-}$	(18 ± 2)%	(4.1 ± 0.5)%	0.9 ± 0.1	9.9 ± 0.2	5.5 ± 0.5	41.9 ± 7.5	232 ± 36
HT-Eed	HT-Eed in wild-type	(16 ± 3)%	(4.3 ± 0.1)%	0.9 ± 0.2	9.5 ± 0.1	4.6 ± 0.4	45.2 ± 4.1	214 ± 6
	HT-Eed in *Eed*$^{-/-}$	(18 ± 1)%	(4.1 ± 0.1)%	1.1 ± 0.2	9.2 ± 0.2	5.6 ± 0.4	36.7 ± 3.7	220 ± 11
Control experiments	HT-Cbx7 in wild-type	(27 ± 1)%	(12.9 ± 0.6)%	0.8 ± 0.1	9.4 ± 0.1	3.1 ± 0.2	19.9 ± 2.1	64 ± 5
	HT-NLS in wild-type	100%	NA	0.7 ± 0.1	NA	NA	NA	NA

Supplementary Table 3 (Relative to Figure 3). Kinetic fractions and diffusion constants.

		F_1	D_1 ($\mu m^2 s^{-1}$)	F_2	D_2 ($\mu m^2 s^{-1}$)	F_3	D_3 ($\mu m^2 s^{-1}$)
HT-Ezh2 and its variants	HT-Ezh2 in wild-type	(23 ± 1)%	0.0001	(23 ± 4)%	0.54 ± 0.09	(54 ± 5)%	4.2 ± 0.4
	HT-Ezh2 in *Eed*$^{-/-}$	(24 ± 1)%	0.0001	(40 ± 5)%	0.62 ± 0.02	(36 ± 1)%	4.2 ± 0.2
	HT-Ezh2$^{\Delta SANT2}$ in wild-type	(22 ± 2)%	0.0001	(29 ± 2)%	0.54 ± 0.05	(50 ± 2)%	4.2 ± 0.4

Supplementary Table 4 (Relative to Figure 3). Search mechanism parameters.

		F_{1tb}	F_{1sb}	τ_{tb} (s)	τ_{sb} (s)	N_{trial}	τ_{3D} (s)	τ_{search} (s)
HT-Ezh2 and its variants	HT-Ezh2 in wild-type	(18 ± 1)%	(4.6 ± 0.3)%	0.9 ± 0.1	10.0 ± 0.2	5.0 ± 0.4	41.6 ± 5.2	210 ± 19
	HT-Ezh2 in *Eed*$^{-/-}$	(18 ± 2)%	(4.8 ± 0.5)%	0.9 ± 0.2	6.9 ± 0.1	4.7 ± 0.4	28.7 ± 5.1	138 ± 22
	HT-Ezh2$^{\Delta SANT2}$ in wild-type	(20 ± 2)%	(1.9 ± 0.2)%	0.7 ± 0.1	5.6 ± 0.2	11.3 ± 0.8	24.8 ± 2.8	287 ± 24

Supplementary Table 5 (Relative to Figure 4). Kinetic fractions and diffusion constants.

		F_1	D_1 ($\mu m^2 s^{-1}$)	F_2	D_2 ($\mu m^2 s^{-1}$)	F_3	D_3 ($\mu m^2 s^{-1}$)
HT-Ezh2/F-H3.3 in HEK293T	5.0 ms	(18 ± 1)%	0.01	(30 ± 3)%	0.35 ± 0.05	(52 ± 3)%	4.5 ± 0.8
	10.0 ms	(18 ± 1)%	0.005	(29 ± 3)%	0.56 ± 0.06	(53 ± 2)%	4.2 ± 0.3
	20.0 ms	(21 ± 1)%	0.002	(30 ± 2)%	0.53 ± 0.03	(49 ± 1)%	4.2 ± 0.8
	30.0 ms	(23 ± 4)%	0.0001	(29 ± 1)%	0.58 ± 0.01	(48 ± 4)%	4.4 ± 0.4
HT-Ezh2/F-H3.3K27M in HEK293T	5.0 ms	(17 ± 1)%	0.01	(26 ± 1)%	0.42 ± 0.20	(57 ± 2)%	4.4 ± 0.1
	10.0 ms	(21 ± 1)%	0.001	(27 ± 4)%	0.56 ± 0.06	(52 ± 4)%	4.4 ± 0.4
	20.0 ms	(22 ± 2)%	0.0001	(26 ± 2)%	0.57 ± 0.04	(52 ± 2)%	4.0 ± 0.3
	30.0 ms	(23 ± 3)%	0.0001	(22 ± 6)%	0.55 ± 0.05	(55 ± 5)%	4.4 ± 0.1
HT-Cbx7/F-H3.3 in HEK293T	5.0 ms	(39 ± 1)%	0.01	(30 ± 1)%	0.50 ± 0.02	(31 ± 3)%	4.1 ± 1.2
	10.0 ms	(40 ± 1)%	0.0005	(28 ± 5)%	0.42 ± 0.02	(32 ± 5)%	4.4 ± 0.9
	20.0 ms	(44 ± 1)%	0.0001	(29 ± 1)%	0.52 ± 0.08	(27 ± 2)%	3.4 ± 0.2
	30.0 ms	(46 ± 1)%	0.0001	(27 ± 2)%	0.50 ± 0.09	(27 ± 2)%	4.4 ± 0.4
HT-Cbx7/F-H3.3K27M in HEK293T	5.0 ms	(28 ± 3)%	0.009	(44 ± 6)%	0.38 ± 0.04	(28 ± 8)%	4.1 ± 1.2
	10.0 ms	(32 ± 1)%	0.005	(40 ± 2)%	0.38 ± 0.02	(28 ± 3)%	4.0 ± 0.6
	20.0 ms	(32 ± 1)%	0.003	(38 ± 3)%	0.44 ± 0.15	(30 ± 2)%	3.7 ± 0.4
	30.0 ms	(32 ± 2)%	0.0001	(38 ± 7)%	0.47 ± 0.02	(30 ± 8)%	3.9 ± 0.1

Supplementary Table 5 continued.

		F_1	D_1 ($\mu m^2 s^{-1}$)	F_2	D_2 ($\mu m^2 s^{-1}$)	F_3	D_3 ($\mu m^2 s^{-1}$)
HT-Ezh2/F-H3.3 in 9427	5.0 ms	(18 ± 2)%	0.007	(29 ± 3)%	0.50 ± 0.14	(53 ± 1)%	4.4 ± 0.2
	10.0 ms	(18 ± 1)%	0.009	(27 ± 6)%	0.77 ± 0.08	(55 ± 6)%	4.6 ± 0.5
	20.0 ms	(22 ± 3)%	0.005	(28 ± 3)%	0.63 ± 0.07	(50 ± 1)%	3.8 ± 0.4
	30.0 ms	(21 ± 2)%	0.0001	(23 ± 7)%	0.61 ± 0.11	(56 ± 7)%	4.2 ± 0.5
HT-Ezh2/F-H3.3K27M in SF8628	5.0 ms	(17 ± 1)%	0.01	(27 ± 3)%	0.39 ± 0.05	(56 ± 4)%	4.6 ± 0.4
	10.0 ms	(18 ± 1)%	0.0001	(27 ± 5)%	0.55 ± 0.02	(55 ± 2)%	4.2 ± 0.1
	20.0 ms	(19 ± 1)%	0.008	(29 ± 1)%	0.59 ± 0.05	(52 ± 3)%	4.4 ± 0.2
	30.0 ms	(22 ± 2)%	0.0001	(23 ± 5)%	0.61 ± 0.07	(55 ± 2)%	4.6 ± 0.2
HT-Cbx7/F-H3.3 in 9427	5.0 ms	(39 ± 5)%	0.004	(35 ± 3)%	0.44 ± 0.01	(26 ± 3)%	4.7 ± 0.1
	10.0 ms	(41 ± 3)%	0.002	(34 ± 2)%	0.45 ± 0.09	(25 ± 3)%	4.4 ± 0.3
	20.0 ms	(42 ± 4)%	0.0001	(29 ± 1)%	0.45 ± 0.02	(29 ± 5)%	3.5 ± 0.5
	30.0 ms	(42 ± 1)%	0.0001	(31 ± 3)%	0.50 ± 0.14	(27 ± 2)%	4.4 ± 0.4
HT-Cbx7/F-H3.3K27M in SF8628	5.0 ms	(30 ± 3)%	0.01	(36 ± 3)%	0.42 ± 0.05	(34 ± 2)%	4.5 ± 0.5
	10.0 ms	(31 ± 3)%	0.004	(36 ± 3)%	0.43 ± 0.06	(33 ± 5)%	4.0 ± 0.3
	20.0 ms	(32 ± 3)%	0.004	(36 ± 2)%	0.50 ± 0.04	(32 ± 3)%	4.1 ± 0.4
	30.0 ms	(30 ± 3)%	0.0001	(35 ± 5)%	0.53 ± 0.08	(35 ± 3)%	4.1 ± 0.1

Supplementary Table 6 (Relative to Figure 5). Search mechanism parameters.

	F_{1tb}	F_{1sb}	τ_{tb} (s)	τ_{sb} (s)	N_{trial}	τ_{3D} (s)	τ_{search} (s)
HT-Ezh2/F-H3.3 in HEK293T	(19 ± 4)%	(4.8 ± 1.0)%	1.0 ± 0.1	10.2 ± 0.2	5.0 ± 0.4	40.0 ± 8.6	204 ± 40
HT-Ezh2/F-H3.3K27M in HEK293T	(18 ±3)%	(5.2 ± 0.7)%	1.0 ± 0.1	15.3 ± 0.2	4.4 ± 0.4	62.6 ± 8.5	278 ± 30
HT-Cbx7/F-H3.3 in HEK293T	(35 ± 1)%	(11.8 ± 0.3)%	1.0 ± 0.1	9.7 ± 0.1	4.0 ± 0.3	17.7 ± 1.5	73 ± 3
HT-Cbx7/F-H3.3K27M in HEK293T	(25 ± 1)%	(7.0 ± 0.4)%	1.1 ± 0.1	9.9 ± 0.1	4.6 ± 0.4	27.8 ± 3.0	133 ± 10
HT-Ezh2 in 9427	(17 ± 2)%	(4.4 ± 0.4)%	0.9 ± 0.1	8.7 ± 0.1	4.9 ± 0.3	37.8 ± 3.8	188 ± 12
HT-Ezh2 in SF8628	(17 ± 1)%	(4.3 ± 0.3)%	1.0 ± 0.1	11.8 ± 0.2	3.4 ± 0.4	76.2 ± 10.9	265 ± 24
HT-Cbx7 in 9427	(32 ± 1)%	(10.1 ± 0.3)%	1.0 ± 0.1	8.5 ± 0.1	4.2 ± 0.3	17.5 ± 1.6	76 ± 4
HT-Cbx7 in SF8628	(23 ± 2)%	(7.1 ± 0.6)%	1.1 ± 0.1	8.5 ± 0.1	3.9 ± 0.3	28.1 ± 4.1	119 ± 15

Supplementary Table 7 (Relative to Figure 6). Kinetic fractions and diffusion constants.

		F_1	D_1 ($\mu m^2 s^{-1}$)	F_2	D_2 ($\mu m^2 s^{-1}$)	F_3	D_3 ($\mu m^2 s^{-1}$)
HT-Ezh2^{Catalytic}	H3.3-FLAG	(17 ± 3)%	0.0001	(24 ± 2)%	0.45 ± 0.06	(59 ± 3)%	4.7 ± 0.1
	H3.3K27M-FLAG	(18 ± 2)%	0.0001	(24 ± 6)%	0.53 ± 0.07	(58 ± 5)%	4.7 ± 0.2

Supplementary Table 8 (Relative to Figure 6). Search mechanism parameters.

		F_{1tb}	F_{1sb}	τ_{tb} (s)	τ_{sb} (s)	N_{trial}	τ_{3D} (s)	τ_{search} (s)
HT-Ezh2^{Catalytic}	H3.3-FLAG	(14 ± 3)%	(2.8 ± 0.5)%	0.8 ± 0.1	4.7 ± 0.1	6.1 ± 0.4	25.8 ± 4.4	162 ± 25
	H3.3K27M-FLAG	(15 ± 2)%	(2.6 ± 0.3)%	0.8 ± 0.1	7.3 ± 0.2	6.9 ± 0.5	39.6 ± 4.6	277 ± 24

Supplementary Table 9 (Relative to Figure 8). Kinetic fractions and diffusion constants.

	Dox	F_1	F_1^N	D_1 ($\mu m^2 s^{-1}$)	F_2	D_2 ($\mu m^2 s^{-1}$)	F_3	D_3 ($\mu m^2 s^{-1}$)
HT-Cbx7 in SF8628	0.1 µg per ml	(30 ± 3)%	1.0 ± 0.1	0.0001	(35 ± 5)%	0.53 ± 0.08	(35 ± 3)%	4.1 ± 0.1
	1.0 µg per ml	(32 ± 3)%	1.6 ± 0.2	0.0001	(37 ± 4)%	0.47 ± 0.10	(31 ± 1)%	4.0 ± 0.6
	2.0 µg per ml	(35 ± 4)%	2.3 ± 0.3	0.0001	(27 ± 1)%	0.54 ± 0.01	(38 ± 3)%	4.2 ± 0.1

F_1^N denotes the double normalization by the relative HT-Cbx7 concentration and the F_1 value at the 0.1 µg per ml of Dox.

Supplementary Table 10 (Relative to Figure 8). Search mechanism parameters.

	Dox	F_{1tb}	F_{1tb}^N	F_{1sb}	F_{1sb}^N	τ_{tb} (s)	τ_{sb} (s)	N_{trial}	τ_{3D} (s)	τ_{search} (s)
HT-Cbx7 in SF8628	0.1 µg per ml	(23 ± 2)%	1.0 ± 0.1	(7.8 ± 0.6)%	1.0 ± 0.1	1.0 ± 0.1	8.5 ± 0.1	3.9 ± 0.3	28.1 ± 4.1	119 ± 15
	1.0 µg per ml	(28 ± 3)%	1.8 ± 0.2	(4.5 ± 0.4)%	0.9 ± 0.1	0.8 ± 0.1	13.2 ± 0.4	7.3 ± 0.9	38.2 ± 5.5	283 ± 23
	2.0 µg per ml	(32 ± 4)%	2.7 ± 0.3	(4.2 ± 0.5)%	1.0 ± 0.1	0.7 ± 0.1	16.2 ± 0.3	8.5 ± 0.8	43 ± 5.6	369 ± 35

F_{1tb}^N and F_{1sb}^Ndenote the double normalization by the relative HT-Cbx7 concentration and the corresponding value at the 0.1 µg per ml of Dox.

Supplementary Table 11. U-track parameters used in this research.

**Step 1: Detection**	**Gaussian Mixture-Model Fitting**	**Parameters:**
		Gaussian Standard Deviation= 1.7 pixels
		Camera Bit Depth: 16
		**Local Maxima Detection:**
		Alpha-value for Comparison with Local Background= 0.05
		Do Not Check "Use Rolling Window Time-Averaging"
		Do Not Check "Use Absolute Background"
		Gaussian Fitting at Local Maxima:
		Check "Iterate to Estimate Gaussian Standard Deviation”
		Maximum Number of Iterations=10
		Check "Do Iterative Gaussian Mixture-Model Fitting"
		Alpha values:
		Residuals=0.05 Distance= 0.05
		Amplitude= 0.05 Final= 0
		Input and Output:
		Frames to Use= 1-300 for Population or 1-250 for Residence
**Step 2: Tracking**	**Tracking Parameters**	**Parameters:**
		Problem Dimensionality= 2
		Maximum Gap to Close= 5 Frames for Population or 1 Frames for Residence
		Maximum Length of Track Segments from First Step= 1 Frame
		Check “Do segment merging”
		Check “Do segment splitting”
		Do Not Check " Plot histogram of gap lengths after gap closing”
		Check “Show calculation progress in command line”
		Do Not Check "Export tracking result to matrix format”
		**Cost Functions:**

		**Step 1: frame-to-frame linking:**
		Check “Allow direct motion position progration”
		Check “Allow instantaneous direction reversal”
		Brownian Search Radius (in pixels):
		Lower Bound=1
		Upper Bound= 20 for Population or 10 for Residence
		Multiplication Factor for Brownian Search Radius Calculation= 3
		Check “Use nearest neighbor distance to expand Brownian search radius”
		Number of Frames for Nearest Neighbor Distance Calculation= 20 for Population or 10 for Residence.
		Do Not Check “Plot histogram of linking distances”
		**Step 2: gap closing, merging and splitting:**
		**Brownian + Directed motion models**
		Brownian Search Radius (in pixels):
		Lower Bound=1
		Upper Bound= 20 for Population or 10 for Residence
		Multiplication Factor for Brownian Search Radius Calculation= 3
		Check “Use nearest neighbor distance to expand Brownian search radius”
		Number of Frames for Nearest Neighbor Distance Calculation= 20 for Population and 10 for Residence
		How to expand the Brownian search radius with gap length:
		Scaling Power in Fast Expansion Phase= 0.5
		Scaling Power in slow Expansion Phase= 0.01
		Gap length to transition from Fast to Slow Expansion= 5 for Population or 1 for Residence
		Penalty for Increasing Gap Length= 1.5
		Check “In merging and splitting, consider ratio of intensities before and after merge/split:
		Ratio of Intensity: Min Allowed= 0.5 Max Allowed= 2

		Leave it Blank “Value of search Radius Lower Bound for Merging/Splitting (in pixels)”
		Check “Allow direct motion position propagation”
		Check “Allow instantaneous direction reversal”
		Minimum Track Segment Lifetime for Classification as Linear or Random (in frames) = 5
		Multiplication Factor for Linear Search Radius Calculation= 3
		How to scale the linear motion search radius with time:
		Scaling Power in Fast Expansion Phase= 0.5
		Scaling Power in Slow Expansion Phase= 0.01
		Gap length to transition from Fast to Slow Expansion= 5 for Population or 1 for Residence
		Maximum Angle Between Linear Track Segments (in degree) = 30
		**Kalman Filter Functions**
		Kalman functions= Brownian + Directed motion models
		Parameters:
		Do Not Check “Initial velocity estimate (in pixels/frame)”
		Do Not Check “Reference point for initial velocity estimate (in pixels)”
		Check “None of the two above”
		Leave it Blank “Search Radius for first Iteration (in frames)”
**Step 3: Track analysis**	**Motion Analysis**	**Motion analysis parameters:**
		Problem Dimensionality= 2
		Check “Check and analyze asymmetric tracks”
		Alpha value for asymmetry determination= 0.1
		Alpha value for moment scaling spectrum analysis= 0.05
		Method for calculation the confinement radius: “mean positional standard deviation”

Supplementary Table 12. Spot-On parameters used in this research.

TimeGap = 30
dZ = 0.700
GapsAllowed = 2
TimePoints = 2
BinWidth = 0.010
UseAllTraj = 0
JumpsToConsider = 4
MaxJumpPlotPDF = 1.05
MaxJumpPlotCDF = 3.05
MaxJump = 5.05
SavePlot = 1
DoPlots = 1
ModelFit = 2
DoSingleCellFit = 0
NumberOfStates = 3
FitIterations = 5
FitLocError = 0
FitLocErrorRange = [0.010 0.075]
LocError = 0.035
UseWeights = 0
D_Free1_3State = [1 5]
D_Free2_3State = [0.01 1]
D_Bound_3State = [0.0001 0.01]
