# Supplementary material for: Live-cell single-molecule dynamics of PcG proteins imposed by the DIPG H3.3K27M mutation
Source: Nat Commun. 2018 May 25;9:2080. doi: 10.1038/s41467-018-04455-7 (PMC5970213; doi:10.1038/s41467-018-04455-7)
Supplement: Supplementary file 2 — Description of Additional Supplementary Files [file 41467_2018_4455_MOESM2_ESM.pdf]

Descriptions of Additional Supplementary Files

File Name: Supplementary Movie 1
Description: H2A-HaloTag in wild-type mES cells during 30-ms exposure time.

File Name: Supplementary Movie 2
Description: HaloTag-NLS in wild-type mES cells during 30-ms exposure time.

File Name: Supplementary Movie 3
Description: HaloTag-Cbx7 in wild-type mES cells during 30-ms exposure time.

File Name: Supplementary Movie 4
Description: HaloTag-Ezh2 in wild-type mES cells during 30-ms exposure time.

File Name: Supplementary Movie 5
Description: HaloTag-Eed in wild-type mES cells during 30-ms exposure time.
